# Supplementary material for: Aging and memory of transitional turbulence
Source: Nat Commun. 2025 Sep 26;16:8447. doi: 10.1038/s41467-025-63044-7 (PMC12475086; doi:10.1038/s41467-025-63044-7)
Supplement: Supplementary file 1 — Supplementary Information [file 41467_2025_63044_MOESM1_ESM.pdf]

Supplementary Information For:
Aging and memory of transitional turbulence

Vasudevan Mukund, Chaitanya S. Paranjape, Michael Philip Sitte, Gökhan Yalnız, and Björn Hof*

Institute of Science and Technology Austria (ISTA), 3400 Klosterneuburg, Austria

(Dated: 3rd July 2025)

DISTRIBUTION OF STRIPE LENGTHS

The distribution of the initial stripe lengths, i.e. of the stripe lengths at the beginning of the observation window, is similar for all the different Re in the range investigated, and a typical example at $Re = 640$ is shown in fig. S1. For this range of stripe lengths, the growth (or decay) rate, in accordance with Xiong *et al.*[1], was found to not depend on the initial length of the stripe.

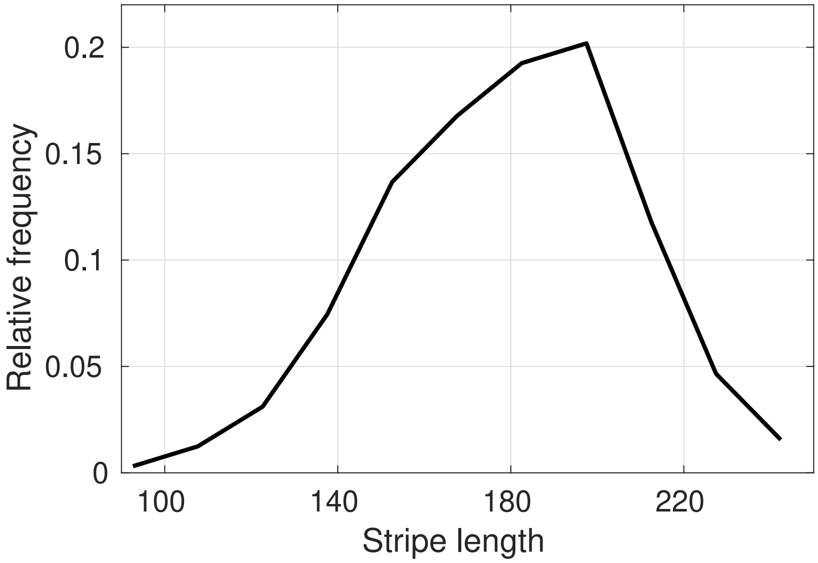

Fig. S1: **Initial Stripe Lengths.** Typical distribution of stripe lengths at the beginning of the observation window, here shown for $Re = 640$.

LARGE- AND SMALL-SCALE FLOW

Several studies[2–5] have pointed out the key role of the large-scale flow in the development and sustenance of turbulent stripes in a variety of wall bounded shear flows. Closely following the treatments in Klotz *et al.*[4], Liu *et al.*[5], we consider the streamwise fluctuation velocity u in the plane $y = 0.5$ and use an isotropic fourth-order Butterworth filter with a cutoff of $\lambda = 10$ to separate the velocity field u into small (u_{SSF}) and large scales (u_{LSF}). We then define the associated energies

$$\begin{aligned} Eu_{\mathrm{LSF}}(t) &= \int_0^{L_z}\int_0^{L_x} u_{\mathrm{LSF}}^2 \mathrm{d}x\mathrm{d}z \\ Eu_{\mathrm{SSF}}(t) &= \int_0^{L_z}\int_0^{L_x} u_{\mathrm{SSF}}^2 \mathrm{d}x\mathrm{d}z\,. \end{aligned} \tag{S1}$$

The time evolution of Eu_{LSF} and Eu_{SSF} for two different runs at Re = 620 are shown in fig. S3. The energy associated with the turbulent small scales initially remains close to constant. However, the energy in the large scales decreases much more rapidly, and once it has decreased by about an order of magnitude, the small scales undergo a rapid decay. Thus, the aging appears to be closely associated with a weakening of the large scale flow, though the precise mechanism involved must be left for future studies.

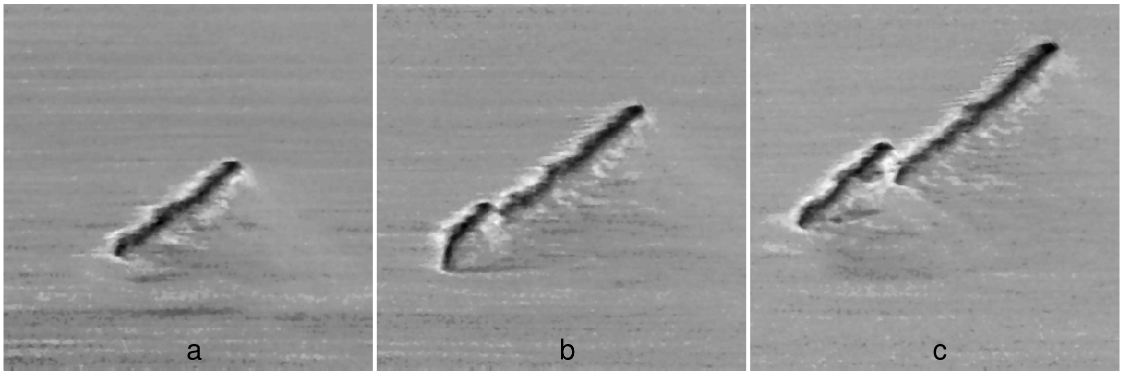

Fig. S2: **Stripe reproduction.** The sequence of three images at Re = 750 shows the fracture of a stripe with a subsequently growing daughter stripe. Consecutive images are 800 advective time units apart. (b) A portion at the upstream tip (trailing edge) breaks off and (c) grows into an independent stripe upstream of the original one.

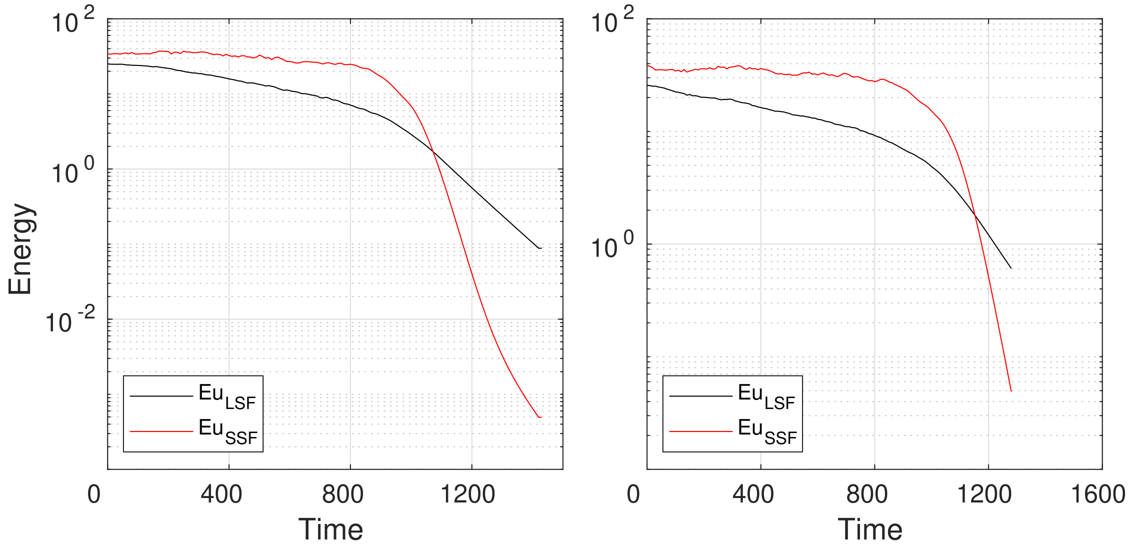

Fig. S3: Time evolution of the energy associated with the large- and small-scales in the flow for two different runs at Re = 620 showing that there is a rapid collapse of the small scales once there is a sufficient decrease in the intensity of the large-scale flow.

GROWTH-FRACTURING CYCLES

A couple of recent computational studies[6,7] investigated transition in channel flow in domain size sufficiently large to capture single stripes (domain sizes were $500h$ streamwise and $250h$ spanwise). Specifically Kanazawa[6] provides the time evolution of stripe lengths at Re $\approx$ 660 (see his fig. 5.1 (a)). The time evolution of stripe lengths can equally be obtained from the supplemental movie by Shimizu and Manneville[7] for Re $\approx$ 700. In order to compare this data to our experiments we compute the distribution of size changes of stripes (for time steps of 100 advective units) as shown in fig. S5. As in our experiments (a) the distributions in simulations (b,c) have a large peak at low positive values which results from the continuous stripe growth encountered at the downstream tip. In contrast, negative events do not have any clear peak and extend to very large values, corresponding to large segments fracturing from the parent stripe. Consequently, the size change distributions of channel flow stripes are strongly asymmetric. This is markedly different from the expected symmetric distributions that can be found for puffs in pipe flow where puff sizes fluctuate around a well-defined average in line with the memoryless nature of puffs.

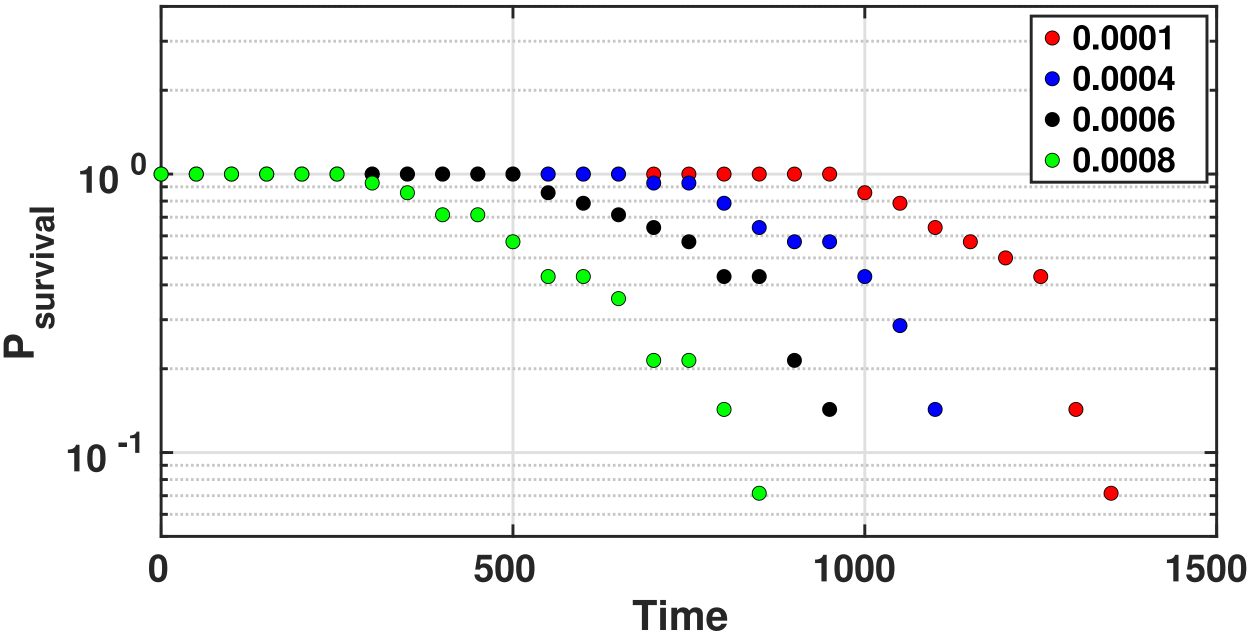

Fig. S4: Survival probability of fully localized stripes at Re = 620. For different values of cutoffs used to determine lifetimes, the qualitative behaviour does not change.

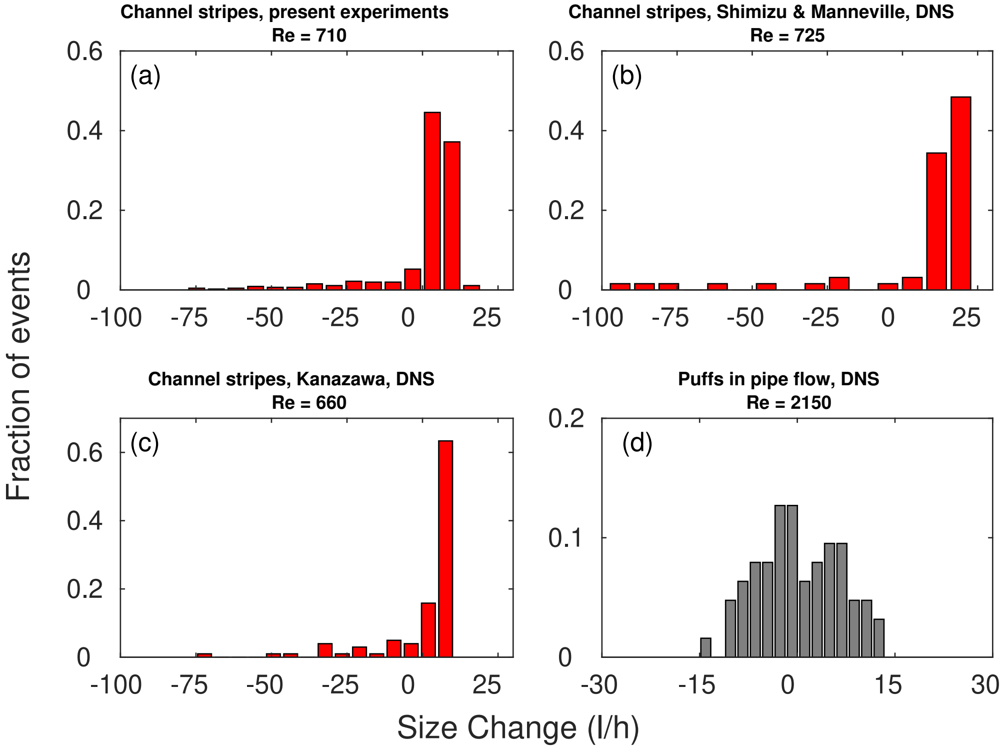

Fig. S5: (a) Distribution of channel flow stripe's length change at time intervals of 100 advective units in experiments and (b) direct numerical simulations of Kanazawa[6] and (c) Shimizu and Manneville[7]. (d) Size changes of puff in pipe flow in contrast show symmetric distributions.

* bhof@ist.ac.at

[1] X. Xiong, J. Tao, S. Chen, and L. Brandt, Turbulent bands in plane-poiseuille flow at moderate reynolds numbers, Phys. Fluids **27**, 041702 (2015).

[2] Y. Duguet and P. Schlatter, Oblique Laminar-Turbulent Interfaces in Plane Shear Flows, Phys. Rev. Lett. **110**, 034502 (2013).

[3] M. Couliou and R. Monchaux, Growth dynamics of turbulent spots in plane couette flow, J. Fluid Mech. **819**, 1 (2017).

[4] L. Klotz, A. M. Pavlenko, and J. E. Wesfreid, Experimental measurements in plane Couette–Poiseuille flow: dynamics of the large- and small-scale flow, J. Fluid Mech. **912**, A24 (2021).

[5] T. Liu, B. Semin, L. Klotz, R. Godoy-Diana, J. E. Wesfreid, and T. Mullin, Decay of streaks and rolls in plane Couette–Poiseuille flow, J. Fluid Mech. **915**, A65 (2021).

[6] T. Kanazawa, *Lifetime and Growing Process of Localized Turbulence in Plane Channel Flow*, Ph.D. thesis, Osaka University (2018).

[7] M. Shimizu and P. Manneville, Bifurcations to turbulence in transitional channel flow, Phys. Rev. Fluids **4**, 113903 (2019).
