# Supplementary material for: Aging and memory of transitional turbulence
Source: Nat Commun. 2025 Sep 26;16:8447. doi: 10.1038/s41467-025-63044-7 (PMC12475086; doi:10.1038/s41467-025-63044-7)
Supplement: Supplementary file 2 — Description of Additional Supplementary Files [file 41467_2025_63044_MOESM2_ESM.pdf]

Description of Additional Supplementary Files

File Name: Supplementary Movie 1

Description: The movie highlights the regular streak creation at the stripe’s downstream tip. In the chosen frame of reference (approximately co-moving with the tip in the streamwise direction), its lateral (/spanwise) translation is easily discernable.
